# Long-term outcomes of penile squamous cell carcinoma in men age ≤50 years old compared with men >50 years old from a single tertiary referral centre: a propensity score matched analysis

**DOI:** 10.1038/s41443-024-00842-5

**Published:** 2024-02-29

**Authors:** Karl H. Pang, Giuseppe Fallara, Morwarid Hemat, Akash Ghosh, Aiman Haider, Alex Freeman, Paul Hadway, Raj Nigam, Rowland Rees, Anita Mitra, Constantine Alifrangis, Asif Muneer, Hussain M. Alnajjar

**Affiliations:** 1 Division of Urology, Department of Surgery, School of Clinical Medicine, The University of Hong Kong, Hong Kong, Hong Kong; 2 Division of Urology, Queen Mary Hospital, Hong Kong, HK Hong Kong; 3 Institute of Andrology, University College London Hospitals NHS Foundation Trust, London, UK; 4 Division of Surgery and Interventional Science, University College London, London, UK; 5 Department of Urology, European Institute of Oncology, Milan, Italy; 6 Department of Histopathology, University College London Hospitals NHS Foundation Trust, London, UK; 7 Department of Urology, Royal Berkshire NHS Foundation Trust, Reading, UK; 8 Department of Urology, Royal Surrey NHS Foundation Trust, Guildford, UK; 9 Department of Urology, University Hospital Southampton NHS Foundation Trust, Southampton, UK; 10 Department of Clinical Oncology, University College London Hospitals NHS Foundation Trust, London, UK; 11 Department of Medical Oncology, University College London Hospitals NHS Foundation Trust, London, UK; 12 Department of Surgical Biotechnology, University College London, London, UK; 13 NIHR Biomedical Research Centre, University College London Hospitals NHS Foundation Trust, London, UK

**Keywords:** Urogenital diseases, Diagnosis

## Abstract

Penile cancer (PeCa) is rare, and the oncological outcomes in younger men are unclear. We aimed to analyse and compare oncological outcomes of men age ≤50 years (y) and >50 years with PeCa. A retrospective analysis of men ≤50 y with penile squamous cell carcinoma managed at a tertiary centre was performed. A propensity score matched cohort of men >50 y was identified for comparison. Matching was according to tumour, nodal stage and the types of primary surgery. Overall survival (OS), disease-specific survival (DSS), recurrence-free survival (RFS), and metastasis-free survivals (MFS) were estimated using Kaplan–Meier plots and compared using log-rank tests. Between 2005–2020, 100 men ≤50 y (median (IQR) age, 46 y (40–49)) were identified and matched with 100 men >50 y (median (IQR) age, 65 y (59–73)). 10, 24, 32, 34 men age ≤50 y were diagnosed in 2005–2007, 2008–2012, 2013–2016 and 2017–2020 respectively. Median (IQR) follow-up was 53.5 (18–96) months. OS at 2 years: ≤50 y, 86%>50 y, 80.6%; 5 years: ≤50 y, 78.1%, >50 y, 63.1%; 10 years: ≤50 y, 72.3%, >50 y, 45.6% (*p* = 0.01). DSS at 2 years: ≤50 y, 87.2%>50 y, 87.8%; 5 years: ≤50 y, 80.9%>50 y, 78.2%; 10 years: ≤50 y, 78%, >50 y, 70.9% (*p* = 0.74). RFS was 93.1% in the ≤50 y group (vs. >50 y, 96.5%) at 2 year, and 90% (vs. >50 y, 88.5%) at 5 years, *p* = 0.81. Within the ≤50 y group, 2 years and 5 years MFS was 93% (vs. >50 y, 96.5%), and 89.5% (vs. >50 y, 92.7%) respectively, (*p* = 0.40). There were no statistical significance in DFS, RFS and MFS in men age ≤50 y and >50 y. PeCa in younger patients is fatal, public awareness and patient education are crucial for early detection and management.

## Introduction

Penile cancer (PeCa) is a rare malignancy and the GLOBOCAN Cancer Statistics estimated 36,068 (0.2%) new cases of PeCa and 13,211 (0.1%) PeCa-related deaths worldwide in 2020 [1]. In the United Kingdom (UK), there were 666 new cases in 2015–2017, and 137 deaths in 2016–2018 [2]. The UK age-standardised incidence rates increased by 21% since the 1970s according to the Office of National Statistics (ONS) [3] and 28% since the 1990s according to Cancer Research UK (CRUK) [2].

Squamous cell carcinoma (SCC) accounts for over 95% of PeCa, and is associated with Human Papillomavirus (HPV) in 30–50% of invasive cases [4, 5]. Being diagnosed with PeCa and undergoing treatment may result in significant physical and psychosexual side effects, especially in younger and sexual active men [6]. PeCa mainly affects men of advanced age with a peak incidence in the 6th to 7th decade [1]. The change in sexual behaviour and HPV infection may account for the rise in PeCa incidence. The incidence of PeCa and oncological details and outcomes of young men under the age of 50 years (y) are unknown. In addition, comparisons of oncological outcomes amongst the younger (≤50 y) and older (>50 y) groups are also unknown.

Here, we report our oncological outcomes of men aged ≤50 y with penile SCC and compare it to a propensity score matched cohort of men aged >50 y with penile SCC from a single tertiary referral centre.

## Patients and Methods

### Patients

Following institutional board registration, a retrospective analysis of data from patients treated for penile SCC between Jan 2005 and Jan 2020 at a single tertiary referral centre was performed. Pathology reports, staging images, operation notes and follow-up data were analysed. There is no age definition for young or older PeCa patients, therefore, an arbitrary cut-off of 50 y was used. A 50 y cut-off was chosen on an arbitrary evaluation based on literature search on cut-offs for young vs. old patients for other urological malignancies [7–9]. The study was reported in accordance with the STROBE checklist (Supplementary Table 1) [10].

#### Management: diagnostic workup

Patients referred for suspected PeCa underwent diagnostic evaluation as per the European Association of Urology (EAU) guidelines [11]. A penile biopsy was performed to confirm the histological diagnosis of PeCa. Disease staging included a penile MRI and a chest and abdominal-pelvic CT. Inguinal lymph nodes were initially assessed by physical examination, ultrasound (US) with or without US-guided fine-needle aspiration (FNA) if lymph nodes were enlarged. Dynamic sentinel lymph node biopsy (DSLNB) was performed in ≥G2pT1 cases and those with non-enlarged lymph nodes on US that were not required to undergo FNA (cN0). Patients were discussed in a supraregional penile cancer multidisciplinary team (sMDT) meeting.

#### Management: primary surgery

Localised disease on the penis was treated by performing one of the following surgical options depending on the location (T-stage) of the disease:

##### Radical Circumcision and Wide local excision (WLE)

A radical circumcision, where the inner foreskin is excised to the coronal sulcus is sufficient if the lesion is confined to the foreskin. In phimotic cases, a dorsal slit is performed first (not incising the tumour) to fully retract the prepuce to examine the glans penis and urethral meatus. The prepuce is removed en-bloc with the lesion via an outer and inner prepuce incision. Small superficial shaft lesions, i.e. not involving the corpus spongiosum or cavernoma may be excised in whole (WLE) and the defect closed with absorbable sutures. If a primary skin closure results causes tension in the fully erect penis, a local skin flap or an extragenital split-thickness skin graft (STSG) may be required.

##### Glansectomy with or without distal corporectomy

Lesions confined to the glans (cT2 disease) are treated with a glansectomy. An incision is made on the outer prepuce, or if the patient is circumcised, an incision is made along the scar. The incision is deepened circumferentially, and a plane is created between above Buck’s fascia and the glans. This is to preserve the vascular supply if a graft is planned. If a STSG is not required, especially if the tumour is adherent to Buck’s fascia, dissection is performed under the fascia. The glans is dissected off the corporal bodies until the urethra is the only remaining attachment, maintaining ~1 cm length beyond the corpora cavernosa if possible. The urethra is transected, spatulated ventrally and is splayed over the corpora cavernosa heads at 2–4 points with 5–0 absorbable suture. Biopsies of the urethral margin and corporal bodies are taken. If the distal corporal bodies are involved macroscopically, distal corporectomies are performed. The skin is brought to the urethra and the shaft skin edges with 5–0 and 4–0 absorbable sutures. A 14Fr 2-way indwelling catheter is left in place for ~12 days.

##### Partial penectomy

Patients with distal disease involving the corpus cavernosa (cT3) are managed with a partial penectomy to preserve penile length for sexual and urinary function. A skin incision is made proximal to the tumour and extended circumferentially down to Buck’s fascia. The dorsal vasculature is ligated and the corpora cavernosa are incised proximal to the tumour with ~5 mm margin. The urethra should be left 10–15 mm longer than the corpora if the lesion is not ventral, and spatulated and centralised over corpora cavernosa with a 5-0 absorbable suture. The shaft skin is subsequently used to cover the stump and sutured to the urethral mucosal edge. A14-16Ch Foley catheter is left in situ and removed after 7 days. A STSG can also be used to avoid the shaft retracting and allow better cosmetic outcome. The main complication of partial penectomy is meatal stenosis. The risk can be minimised by ensuring that the urethral spatulation conducted is of an adequate length, 10–15 mm.

##### Total penectomy/Subtotal penectomy

If the whole shaft of the penis and crura are involved, a total penectomy with perineal urethrostomy is required. An elliptical incision is performed around the tumour, followed by dissection through the skin and superficial fascia around the tumour. Subsequent division and ligation of the deep dorsal vein and neurovascular bundle is performed along with the suspensory ligament. Dissection is carried out beyond the tumour or down to the crura if there is extensive proximal involvement. The crura are isolated with complete division of them onto the ischial tuberosity via a perineal incision. In subtotal penectomy the  corporal body is divided at the penoscrotal junction  and oversewn using a 2/0 PDS suture. The urethra is mobilised off the corporal bodies with adequate length and spatulated ventrally. A inverted ‘U or Y” shape incision is made in the perineum and the urethra is brought out. The perineal skin is sutured to the urethra with 4/0 and 5/0 absorbable sutures. A 14–16Ch Foley catheter is then left insitu and removed after ~7–10 days. A drain is left in the perineum and in the pubic area, with a pressure dressing applied to reduce hematoma formation. The main complication from total/subtotal penectomy is perineal urethrostomy stenosis.

##### Glans resurfacing

This procedure is indicated for PeIN or cT1a disease. The glans skin is marked in quadrants from the urethral meatus to the coronal sulcus. A tourniquet is applied and the tip of each quadrant is lifted, starting at the urethral meatus. The glans epithelium and subepithelium are dissected from the meatus to the coronal sulcus. The tourniquet is removed once all quadrants have been excised. Haemostasis is secured and a STSG is harvested, sutured to the corona sulcus and urethral edge with 4–0 and 5–0 absorbable sutures. Absorbable quilting sutures with monofilament poliglecaprone 5–0 are used. A 14–16ch urethral catheter is left insitu for around 12 days and a paraffin soaked dressing is applied and sutured in place. Coronal-sparing glans resurfacing can also be performed in cases where the coronal ridge and sulcus are not affected, this allows preservation of the coronal ridge helping maintain erogenous sensation, sexual function with excellent cosmetic outcomes [12].

##### Split-thickness skin graft (STSG)

A STSG can be used to construct a neoglans following glans resurfacing, glansectomy or partial penectomy. Commonly, the thigh is selected for STSG harvesting using an air-powered dermatome. Graft thickness ranges from 0.014 to 0.018 inches. In case of smoking, vascular disease or diabetes, graft healing can be compromised. Therefore, the use of grafts should be discouraged in severe vascular disease, uncontrolled diabetes or when the patient refuses to quit smoking. Complications include graft infection or graft failure requiring debridement.

#### Management: lymphadenectomy

Patient with cancer detected in FNA or DSLNB samples were managed with radical inguinal lymphadenectomy (ILND) with or without pelvic lymph node dissection if they had pN2 disease or extracapsular nodal extension. Post-operative histological reports and staging images were reviewed in a sMDT meeting to decide on adjuvant treatment.

### Primary and secondary outcomes

The primary outcomes were overall survival (OS), disease-specific survival (DSS), and secondary outcomes included recurrence-free survival (RFS) and metastasis-free survival (MFS).

### Statistical analysis

A 1:1 propensity score matched cohort of 100 men >50 y with penile SCC based on type of surgical procedure, grade and stage of disease was identified.

A 1:1 propensity score matched cohort (without replacement) was created by matching each patient ≤50 y with 1 > 50 y, by use of nearest neighbour matching within a propensity score-based caliper of 0.20. The 2 cohorts were matched according to clinical local and nodal stage and to the type of surgery of the primary tumour. All subsequent analyses were performed on the matched cohort. Balance was checked after matching for each covariate.

Averages were calculated using the median and interquartile range. Categorical data were analysed using Chi-square test. Non-parametric continuous data were analysed using Mann–Whitney *U* test for comparing 2 groups and Kruskal–Wallis test for comparing more than 2 groups. All tests were 2-tailed. Kaplan–Meier (KM) plots were used to estimate OS, DSS, RFS and MFS and comparisons were performed using the log-rank test. The time to event was calculated from the date of diagnosis. Statistical significance was defined as *p*-value < 0.05. All statistical analyses were performed using SPSS (version 28.0).

## Results

After matching, 100 men with penile SCC aged ≤50 y and 100 men >50 y old were included in the analysis. The median (interquartile range, IQR) age was 46 y (40–49) vs. 65 y (59–73) (*p* < 0.001), for the 2 groups respectively (Table 1). There were no statistically significant differences for the matched variables between the 2 groups, i.e. the propensity score matching was adequately balanced. Overall, the median (IQR) follow-up was 46.5 (15.25–77.5) months for the ≤50 y group and 62 (19.75–115) for the >50 y group.

**Table 1:** Baseline clinical and histopathological characteristics and oncological outcomes of patients ≤50 years and >50 years old.

	Overall	≤50 years	>50 years	*p*-value*
Number of patients, *n*	200	100	100	
Follow-up (months), median (IQR)	53.5 (18–96)	46.5 (15.25–77.5)	62 (19.75–115)	0.04
Age, (median, IQR)	50.5 (46–65)	46 (40–49)	65 (59–73)	<0.001
Primary surgery, *n* (%)				0.09
Radical circumcision	38 (19.0)	25 (25.0)	13 (13.0)	
WLE	34 (17)	12 (12)	22 (22)	
Glansectomy +/− distal corporectomy +/− SSG	91 (45.5)	44 (44)	47 (47)	
Partial penectomy +/− SSG	20 (10)	12 (12)	8 (8)	
Total penectomy	16 (8)	6 (6)	10 (10)	
Glans resurfacing	1 (0.5)	1 (1)	0 (0)	
Grade, *n* (%)				0.75
1	1 (0.5)	1 (1)	0 (0)	
1–2	7 (3.5)	3 (3)	4 (4)	
2	57 (28.5)	26 (26)	31 (31)	
2–3	75 (37.5)	40 (40)	35 (35)	
3	60 (30)	30 (30)	30 (30)	
Histology, *n* (%)				
Sarcomatoid	3 (1.5)	3 (3)	0 (0)	0.08
Basaloid	33 (16.5)	16 (16)	17 (17)	0.85
NOS	92 (46)	50 (50)	42 (42)	0.26
PeIN	84 (42)	46 (46)	38 (38)	0.25
LS	58 (29)	25 (25)	33 (33)	0.21
HPV	17 (8.5)	12 (12)	5 (5)	0.08
pT-stage, *n* (%)				0.18
1	104 (52)	52 (52)	52 (52)	
2	61 (30.5)	30 (30)	31 (31)	
3	28 (14)	17 (17)	11 (11)	
4	7 (3.5)	1 (1)	6 (6)	
pN-stage, *n* (%)				0.37
x	33 (16.5)	12 (12)	21 (21)	
0	110 (55)	59 (59)	51 (51)	
1	25 (12.5)	11 (11)	14 (14)	
2	14 (7)	7 (7)	7 (7)	
3	18 (9)	11 (11)	7 (7)	

*IQR* interquartile range, *WLE* wide local excision, *SSG* split skin graft, *NOS* not otherwise specified, *PeIN* penile intraepithelial neoplasia, *LS* lichen sclerosus, *HPV* Human Papillomavirus.

*Comparison between men age ≤50 years and >50 years. Categorical data were analysed using Chi-square test. Non-parametric continuous data were analysed using Mann–Whitney *U* test.

### Number of men age ≤50 years diagnosed in different year groups

**Fig. 1:**
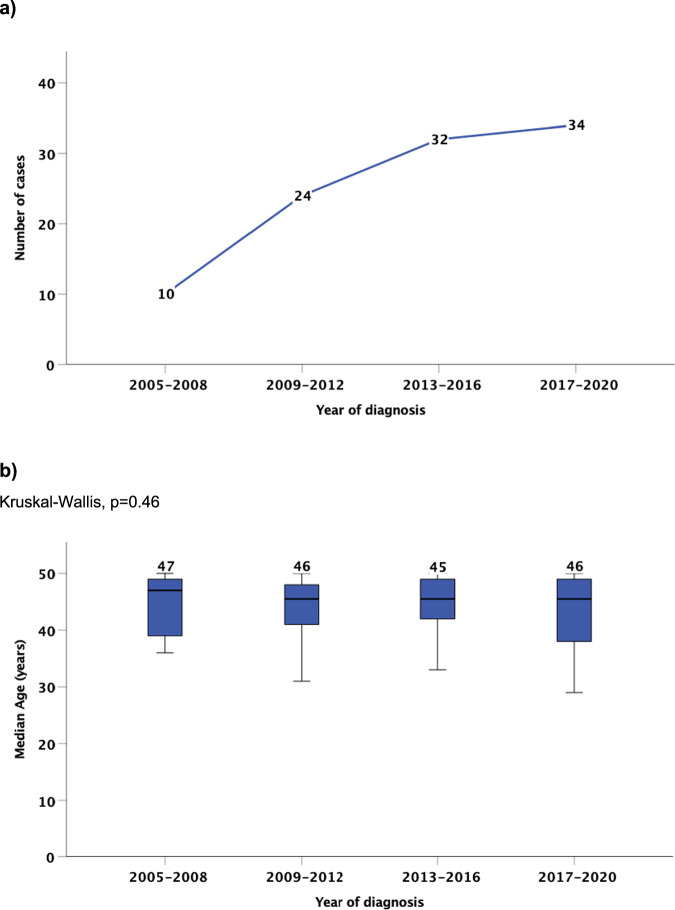
Trends in the diagnosis of penile squamous cell carcinoma in men age ≤50 years. **a** Number of new diagnoses during different year groups. **b** Median age of diagnosis during different year groups.

The number of new cases diagnosed in the year groups 2005–2008, 2009–2012, 2013–2016 and 2017–2020 was 10, 24, 32 and 34 respectively (Fig. 1a). The median (IQR) age of diagnosis in the year groups 2005–2008, 2009–2012, 2013-2016 and 2017–2020 was 47 (39.25–49), 46 (41.5–48), 45 (42–47.5) and 46 (38.5–49) years respectively (*p* = 0.46) (Fig. 1b).

### Baseline clinical and histopathological characteristics of men ≤50 years

Procedures performed included radical circumcision, *n* = 25 (25%); WLE, *n* = 12 (12%); glansectomy with or without distal corporectomy or STSG, *n* = 44 (44%); partial penectomy with or without STSG, *n* = 12 (12%); subtotal/total penectomy with perineal urethrostomy, *n* = 6 (6%); glans resurfacing, *n* = 1 (1%) for men ≤50 y (Table 1).

Pathological T-stage 1, 2, 3 and 4 disease was present in 52 (52%), 30 (30%), 17 (17%) and 1 (1%) respectively for men ≤50 y. Three (3%) patients had sarcomatoid differentiation and 16 (16%) had basaloid differentiation. Lichen sclerosus and HPV were detected in 25 (25%) and 12 (12%) of cases (Table 1). HPV infection was found in 12 (12%) in the ≤50 y cohort and in 5 (5%) in the >50 y cohort (*p* = 0.08).

A total of 88 (88%) patients underwent lymph node surgery (unilateral, *n* = 5; bilateral, *n* = 83) and out of these men, 29 (33%) had positive disease (unilateral, *n* = 16; bilateral, *n* = 13) (Table 1).

### Oncological outcomes

#### Overall and disease-specific survival

**Fig. 2:**
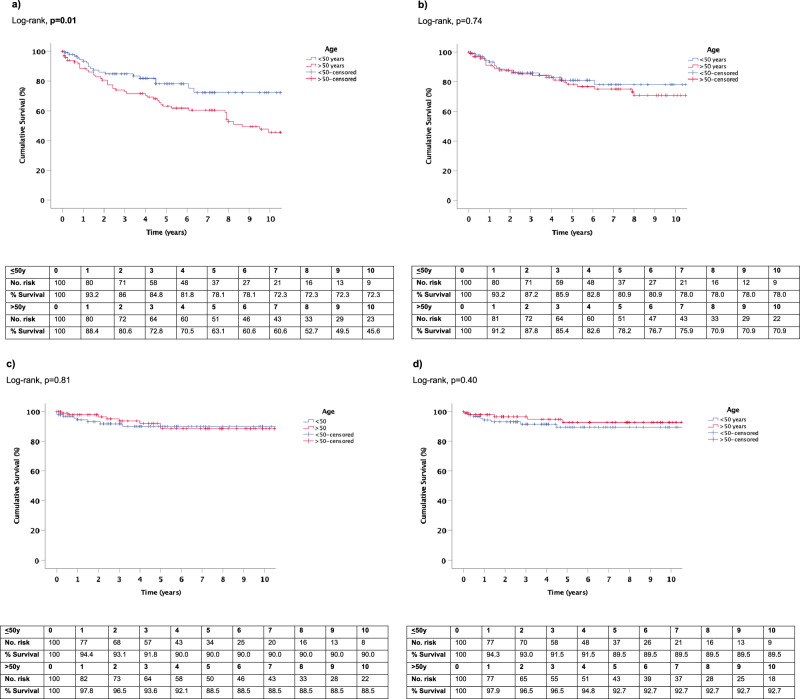
Kaplan–Meier estimates of survival in men age ≤50 years and >50 years. **a** Overall survival. **b** Disease-specific survival. **c** Recurrence-free survival. **d** Metastasis-free survival.

At the time of analysis 80 (80%) men ≤50 y and 51 (51%) men >50 y were alive (*p* < 0.001) (Table 2). Within the ≤50 y group, KM plots predicted a 2 years, 5 years and 10 years OS rate of 86% (vs. >50 y, 80.6%), 78.1% (vs. >50 y, 63.1%) and 72.3% (vs. >50 y, 45.6%) respectively, *p* = 0.01 (Fig. 2a).

**Table 2:** Oncological outcomes of patients ≤50 years and >50 years old.

	Overall	≤50 years	>50 years	*p*-value*
Recurrence, *n* (%)	16 (8)	8 (8)	8 (8)	1.0
Time to recurrence (months), median (IQR)	24 (7–38)	18 (7–37)	32.5 (11.25–48)	0.21
Metastasis, *n* (%)	13 (6.5)	8 (8)	5 (5)	0.39
Time to metastasis (months), median (IQR)	11 (4–33)	10.5 (4–21.75)	18 (1.5–47)	0.74
Overall mortality, *n* (%)	69 (34.5)	20 (20)	49 (49)	<0.001
Time to OM (months), median (IQR)	29 (13–68)	24 (12–56.5)	36 (14.5–95)	0.17
Disease-specific mortality, *n* (%)	40 (20)	17 (17)	23 (23)	0.29
Time to DSM (months), median (IQR)	18 (10–52.75)	16 (9.5–43)	26 (10–57)	0.55

*IQR* interquartile range, *OM* overall mortality, *DSM* disease-specific mortality.

*Comparison between men age ≤50 years and >50 years. Categorical data were analysed using Chi-square test. Non-parametric continuous data were analysed using Mann–Whitney *U* test.

The KM estimates of DSS at 2 years was ≤50 y, 87.2%, >50 y, 87.8%; 5 years was ≤50 y, 80.9%, >50 y, 78.2%; 10 years was ≤50 y, 78%, >50 y, 70.9% (*p* = 0.74) (Fig. 2b).

#### Recurrence-free survival

During the study period, recurrences occurred in 8 (8%) men in the ≤50 y group and 8 (8%) men in the >50 y group, *p* = 1.0 (Table 2). KM plots predicted RFS of 93.1% in the ≤50 y group (vs. >50 y, 96.5%) at 2 year and 90% (vs. >50 y, 88.5%) at 5 years *p* = 0.81 (Fig. 2c).

#### Metastasis-free survival

Overall, 8 (8%) and 5 (5%) men developed metastatic disease in the ≤50 y and >50 y groups respectively, *p* = 0.39 (Table 2). Within the ≤50 y group, KM plots estimated a 2 years and 5 years MFS rate of 93% (vs. >50 y, 96.5%) and 89.5% (vs. >50 y, 92.7%) respectively, *p* = 0.40 (Fig. 2d).

There were no statistically significant differences in survival outcomes (except OS) between men ≤50 y and >50 y with lymph node positive disease (Supplementary Fig. 1).

## Discussion

We report the outcomes of men age ≤50 y with penile SCC managed in a single tertiary centre during a 15-year period. We found that the number of new cases age ≤50 y increased steadily over the study period, this is in keeping with the ONS [3] and CRUK [2] data on the general increase in the diagnosis of PeCa. The increase in incidence may be explained by changes in sexual behaviour and possibly increased detection due to improved sexually-transmitted infection (STI) and cancer awareness. However, this observation may also be related to a change in referral pattern over time and therefore, may not represent a true increase in the incidence of PeCa in men ≤50 y. HPV is associated with up to 50% of PeCa. Having multiple sexual partners, men who have sex with men (MSM) and early age at first sexual intercourse are associated with increased risk of HPV infection [13, 14]. There were more cases of HPV infection in the ≤50 y (12%) group (vs. >50 y, 5%), but this did not reach statistical significance. HPV/p16 expressions were not routinely performed in our centre prior to the most recent 2022 WHO classification of urinary and male genital tumours or before 2020. This may have in turn underestimated the incidence of HPV observed in our cohort. There have been a decline in both genital warts [15] and cervical cancer [16] in young women in England associated with the 2008 HPV vaccination programme. Therefore, the introduction of the 2019 HPV vaccination programme for boys may result in similar effect on the incidence of HPV and PeCa in the future [17].

The majority (82%) of the younger patients in our study underwent penile-preserving procedures, this is the mainstay of treatment for disease limited to the glans penis and the foreskin, in order to preserve urinary and sexual function, and has been shown to be associated with satisfactory cosmetic outcomes, without compromising oncological outcomes [18–20]. Preserving the penis and sexual function is vital in sexually active patients, particularly in the younger age group.

The 5 years OS was higher for patients ≤50 y compared to >50 y, 78.1% vs. 63.1% (*p* < 0.001), respectively, but the DSS at 5 years was similar, ≤50 y, 80.9% vs. >50 y 78.2% (*p* = 0.74), respectively. This is not a surprise, representing that older patient died more from other causes compared to younger adults, who were more likely to be fitter and healthier. However, we do not have data on co-morbidities and Charlson-score to support this assumption.

Despite matching for clinical stage, the histological features and tumour grades were similar between men aged ≤50 y and those aged >50 y. This means that tumour characteristics are similar in the case of PeCa in young and older adults. Overall, this imply that PeCa can be fatal in all age groups, including young patients. Therefore, clinicians should not underestimate the aggressiveness of PeCa in young patients and research and management should focus on preventing PeCa and early detection [21]. Public awareness and patient education are important and this could be delivered possibly through schools, social media, men’s magazines, billboards, and charity awareness campaigns.

Veeratterapillay et al. [22], reported the oncological outcomes from 203 patients and at a median follow-up of 61 months, the recurrence rate (10.8%) and DSS at 5 years (85%) and 10 years (81%) were similar to data reported in our study.

Overall, PeCa in young adults is an under-investigated field, despite observations of increase in its incidence [2, 3]. Key clinical questions regarding patients’ management in the case of a young age should focus on the optimal surgical approach, where penile-preserving surgery should be preferred in order to preserve quality of life [18] and reduce complications, in particular regarding ILND. The ongoing VELRAD randomised-controlled trial aim to compare the outcomes of laparoscopic versus open ILND [23]. In addition, reconstruction and the optimal duration of follow-up in young men should take into account potential sexual and onco-fertility issues [24, 25].

### Limitations

This study has a few limitations. The retrospective nature of the study may not have captured all the ≤50 y men managed at our centre. Being a tertiary centre, after primary surgery and a period of surveillance, patients are often discharged back to their local urology units, making it difficult to obtain long-term follow-up data. Our study spanned over 15 years and there have been a change in practice over the years such as the use of DSLNB [26], reduced excision margins [27] and increased adoption of penile-preserving surgery [18]. In addition, despite matching, some unmeasured and unbalanced confounders might have affected the results, in turn, may have affected our diagnostic rate and recurrence and survival outcomes.

## Conclusion

Within our cohort, the number of men aged ≤50 y diagnosed with penile SCC increased over the past 15 years. The DSS, RFS and MFS are similar in the ≤50 y and >50 y groups. The overall survival was higher in the younger age group. PeCa in younger patients is just as fatal, public awareness and patient education is crucial for disease prevention, early detection and management.

## Supplementary information

Supplementary Table 1

Supplementary Figures

## Data Availability

All data generated or analysed during this study are included in this published article [and its supplementary information files].
