# Supplementary material for: Long-term outcomes of penile squamous cell carcinoma in men age ≤50 years old compared with men >50 years old from a single tertiary referral centre: a propensity score matched analysis
Source: Int J Impot Res. 2024 Feb 29;37(9):771–7. doi: 10.1038/s41443-024-00842-5 (PMC12474540; doi:10.1038/s41443-024-00842-5)

**Supplementary Figure 1.** Kaplan-Meier estimates of survival in men age <50 years and >50 years with lymph node positive disease.

a) Overall survival; b) Disease-specific survival; c) Recurrence-free survival; d) Metastasis-free survival.

**a)**

Log-rank, p=0.60

**
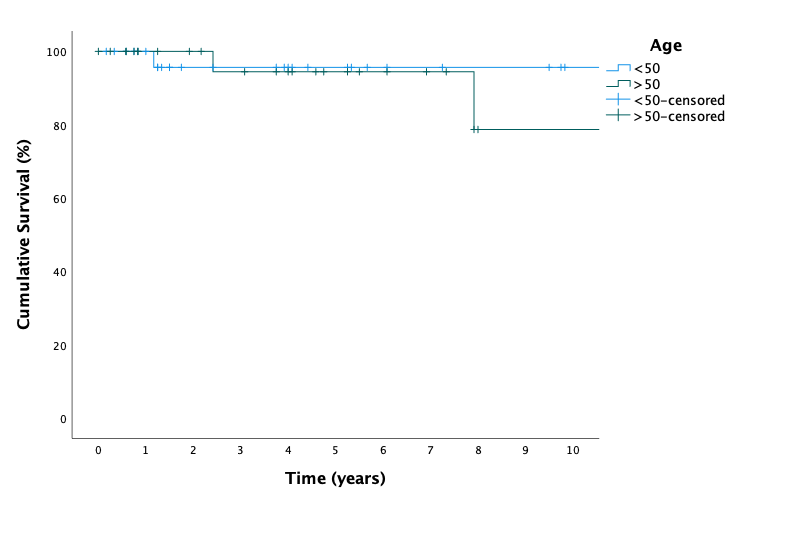
**

**b)**

Log-rank, p=0.53


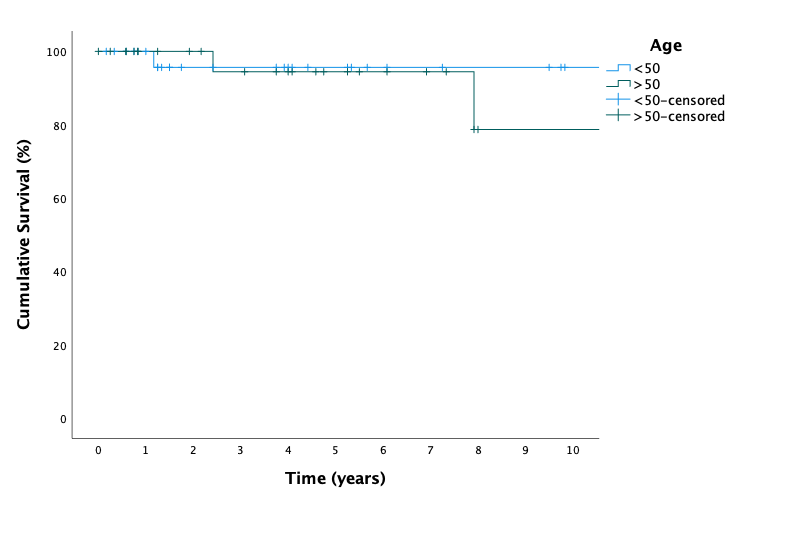


**c)**

Log-rank, p=0.57

**
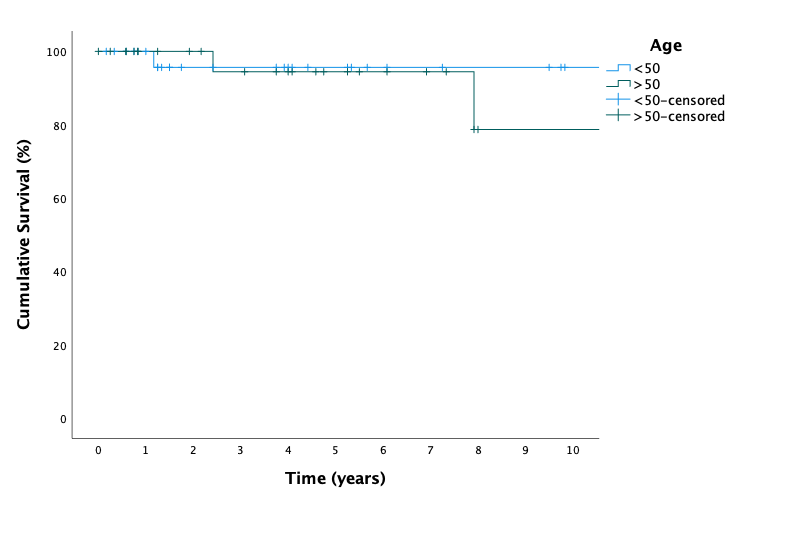
**

**d)**

Log-rank, p=0.77


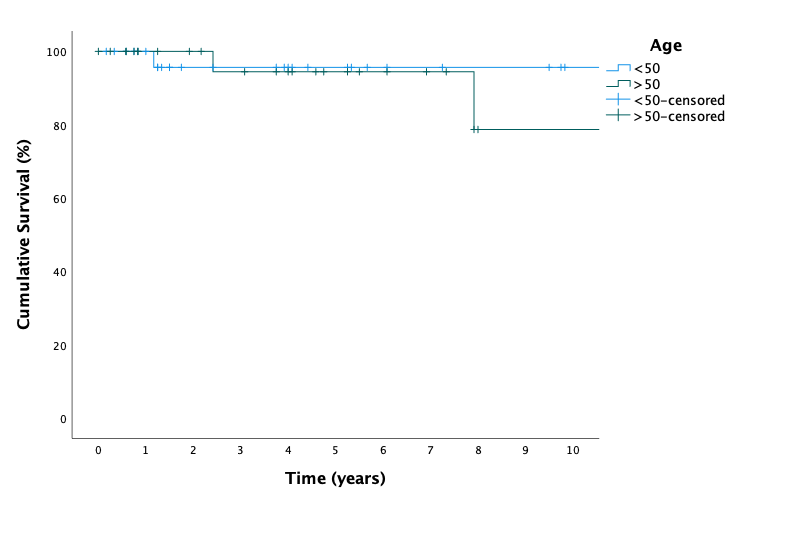

Supplement: Supplementary file 2 — Supplementary Figures [file 41443_2024_842_MOESM2_ESM.docx]
